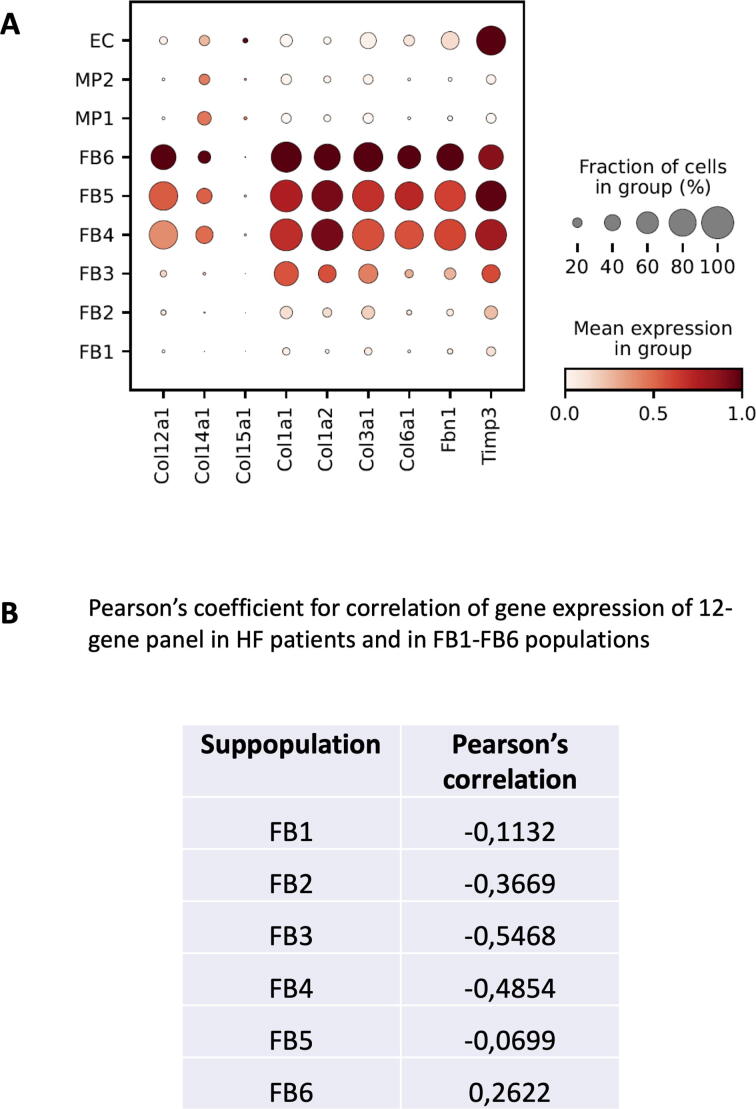# Cardiac fibroblast sub-types *in vitro* reflect pathological cardiac remodeling *in vivo*

**DOI:** 10.1016/j.mbplus.2022.100113

**Published:** 2022-06-06

**Authors:** Kate Møller Herum, Guangzheng Weng, Konstantin Kahnert, Rebekah Waikel, Greg Milburn, Autumn Conger, Paul Anaya, Kenneth S. Campbell, Alicia Lundby, Kyoung Jae Won, Cord Brakebusch

**Affiliations:** a Biotech Research and Innovation Centre (BRIC), University of Copenhagen, 2200 Copenhagen, Denmark; b Department of Biomedical Sciences, Faculty of Health and Medical Sciences, University of Copenhagen, Copenhagen, Denmark; c Department of Physiology, University of Kentucky, Lexington, KY, USA; d Division of Cardiovascular Medicine, University of Kentucky

**Keywords:** Fibrosis, Myofibroblast, Heart failure

## Abstract

Many heart diseases are associated with fibrosis, but it is unclear whether different types of heart disease correlate with different subtypes of activated fibroblasts and to which extent such diversity is modeled during *in vitro* activation of primary cardiac fibroblasts. Analyzing the expression of 82 fibrosis related genes in 65 heart failure (HF) patients, we identified a panel of 12 genes clearly distinguishing HF patients better from healthy controls than measurement of the collagen-related hydroxyproline content. A subcluster enriched in ischemic HF was recognized, but not for diabetes, high BMI, or gender. Single-cell transcriptomic analysis of *in vitro* activated mouse cardiac fibroblasts distinguished 6 subpopulations, including a contractile Acta2^high^ precursor population, which was predicted by time trajectory analysis to develop into Acta2^low^ subpopulations with high production of extracellular matrix molecules. The 12 gene profile identified in HF patients showed highest similarity to the fibroblast subset with the strongest expression of extracellular matrix molecules. Population markers identified were furthermore able to clearly cluster different disease stages in a murine model for myocardial infarct. These data suggest that major features of cardiac fibroblast activation in heart failure patients, in murine heart disease models*,* and in cell culture of primary murine cardiac fibroblast are shared.

## Highlights

• A panel of 12 fibrosis related genes clearly identified heart failure (HF) patients better than measurement of the collagen-related hydroxyproline content.
• A subcluster enriched for ischemic HF was recognized, but not for diabetes, obesity, or gender.
• Single-cell transcriptomic analysis of *in vitro* differentiated mouse cardiac fibroblasts distinguished 6 subpopulations, including a contractile Acta2high precursor population, and Acta2low subpopulations with high production of extracellular matrix molecules.
• The 12 gene profile identified in HF patients showed highest similarity to the fibroblast subset with the strongest expression of extracellular matrix molecules.
• Major features of cardiac fibroblast differentiation in heart failure patients, in murine heart disease models*,* and in cell culture of primary murine cardiac fibroblast are shared.

## Introduction

There is a strong correlation between fibrosis and heart failure [[1], [2]]. Myocardial stiffening caused by cardiac fibrosis is likely responsible for development of heart failure with preserved ejection fraction as well as to contribute to heart failure with reduced ejection fraction. Despite this, no drugs targeting pro-fibrotic activity of cardiac fibroblasts are currently applied in the clinic. Challenges faced when targeting fibrosis include specifically preventing unwanted detrimental effects of fibrosis such as myocardial stiffening, without compromising the integrity of the collagen fiber network required as structural support for the contracting heart muscle cells. Furthermore, fibrosis may present in different forms, mechanically and compositionally, at different stages and types of disease, and the course of fibrosis development may vary even within patients suffering from the same disease type. Several research groups have recently shown that multiple cardiac fibroblast sub-types exist in the healthy and diseased heart [[3], [4], [5], [6], [7]]. Although functional studies of these subtypes are currently lacking, analyses of the gene expression profiles suggest both pro- and anti-fibrotic roles. Thus, for successful targeting, it will be essential to more closely define and understand the pathophysiological heterogeneity of patients with heart failure. We here examine whether the myocardial expression pattern of 82 fibrosis-related genes can be used to group end-stage heart failure patients and whether the groups are differentially associated with clinical parameters such as ischemia and diabetes.

If cardiac fibroblasts are heterogenous *in vivo*, how is this heterogeneity reflected in conventional cell culture of primary mouse fibroblasts? Are fibroblast subpopulations observed *in vitro* and, if yes, do they correlate with fibrosis subtypes of heart-failure patients? Recently, we showed that myofibroblast features, such as smooth muscle alpha actin (*Acta2*) fibers and expression of genes involved in extracellular matrix (ECM) remodeling (*Col1a1* and *Lox*), started to decline after 9–12 days in culture [8], suggesting that fibroblast activation *in vitro* is a dynamic process. To gain more insight into fibroblast activation *in vitro*, we here perform single cell RNA sequencing (scRNAseq) and cell trajectory analysis of primary cardiac fibroblasts from mouse left ventricles that have been cultured on plastic for 15 days and reveal several distinct cardiac fibroblast sub-types and activation paths. Finally, we identify an activated fibroblast subtype *in vitro* with high similarity to the fibrosis gene expression profile observed in heart failure patients.

## Results

### Patients with heart failure can be identified by cardiac gene expression of a panel of 82 fibrosis-related genes

**Fig. 1:**
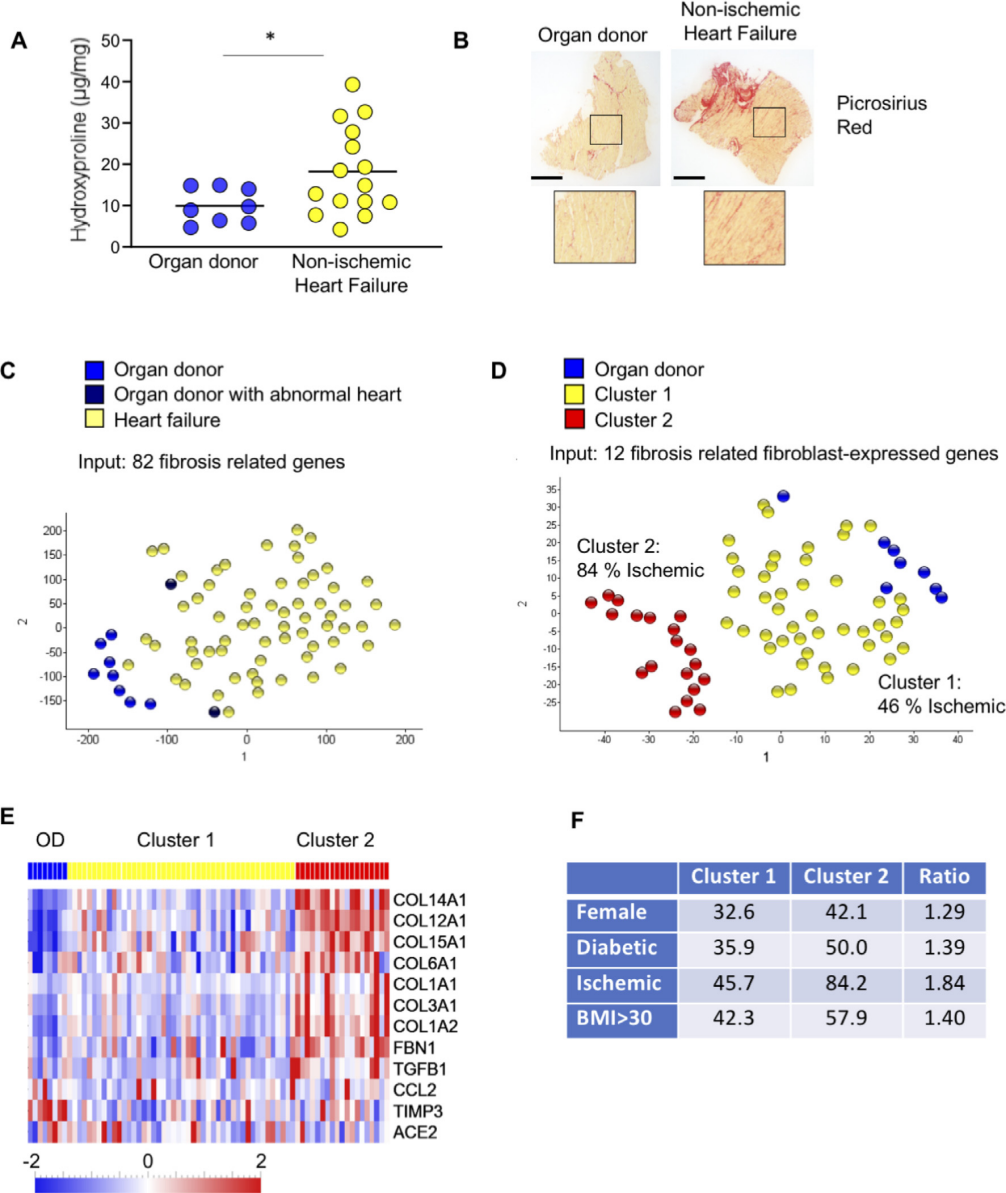
12-gene expression profile distinguishes hearts from HF patients and organ donors. A) Hydroxyproline content of hearts of organ donors and non-ischemic HF patients (n:8/15; *: p less than 0.05). B) Picrosirius Red staining of heart sections of organ donors and non-ischemic HF patients (size bar 2 mm). C) tSNE plot of donors and HF hearts with expression of 82 fibrosis related genes as input. D) tSNE of plot of donors and HF hearts with expression of 12 fibrosis related genes expressed in fibroblasts as input. E) Heat map of donors and HF hearts for expression of 12 fibrosis related genes in fibroblasts. Color code indicates expression level relative to mean expression of gene across patients, red color indicates increased and blue color decreased expression. F) Table indicating the percentage of HF patients in cluster 1 and 2 with specific clinical features. The ratio indicates the enrichment of patients with these features in cluster 2. (For interpretation of the references to color in this figure legend, the reader is referred to the web version of this article.)

End-stage non-ischemic heart failure (HF), the underlying cause of 37 of the 63 HF patients Suppl. Table 1), is characterized by myocardial fibrosis as illustrated in Fig. 1 by hydroxyproline data representing total collagen content (Fig. 1A) and picrosirius staining (Fig. 1B). However, the interindividual variation of the hydroxyproline measurements was high and several HF samples showed levels similar to those of the organ donors. Similarly, picrosirius red staining indicated quite variable fibrosis levels within a section, which may lead to variation in results depending on the sampling region. As an alternative strategy to evaluate fibrosis, we examined myocardial mRNA levels of 82 genes related to fibrotic heart disease (Suppl. Table 2) in tissue from the mid-wall region of the left ventricle in all 65 heart failure patients and 10 organ donor controls (Suppl. Table 1). Genes were selected based on a literature search using the key words “fibrosis”, “heart”, “cardiovascular”, “myocardium”, “myocyte”, as well as variations of these terms, to primarily identify genes that were known to be expressed in the myocardium and could influence cardiac fibrosis, which included known inflammation genes. Both human and rodent model studies were utilized to compile the initial list of genes of interest. Given reports of sex differences in cardiac fibrosis, we also included some sex specific genes which may have had limited evidence for a role in fibrosis, yet activate pathways known to contribute to fibrosis. Lastly, results of a pilot microarray comparing gene expression in the myocardium of human failing hearts to that of non-failing hearts were also used to identify some possible genes of interests. The resulting list of 82 genes included a range of genes, from those with strong evidence for a role in cardiac fibrosis to those with very limited evidence, allowing for the characterization of well-known fibrotic genes, as well as discovery of new players in cardiac fibrosis.

The evaluation of these 82 genes separated all but two heart failure patients from organ donor controls in a tSNE plot (Fig. 1C). Clinical inspection revealed that these two donors that did not segregate with the control group had abnormal heart function (large wall motion abnormality) and geometry (severe concentric hypertrophy) as assessed by echocardiography, demonstrating that fibrotic gene expression can identify patients with heart disease. The two organ donors with abnormal hearts were removed from all subsequent analyses. Thus, hearts of end-stage HF patients have a characteristic expression profile of fibrosis-related genes, which is different from healthy hearts. However, no clear subgrouping within the heart-failure patients was observed.

### Expression profiles of 12 cardiac fibroblast expressed genes identifies sub-groups of heart failure patients

**Supplementary figure 1:**
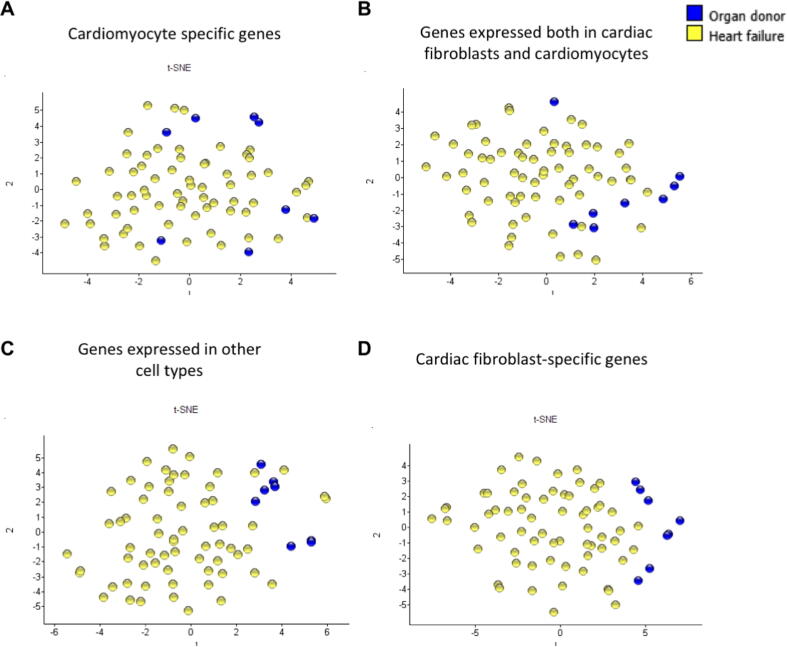


To better understand which cardiac cell types contributed to the fibrotic gene expression profile characteristic for heart failure patients, we used a published human heart single nucleus RNA sequencing data set from Tucker et al. [9], to assign the 82 genes investigated by us to individual cell types. Some transcripts were predominantly expressed in cardiomyocytes (24 genes), some in cardiac fibroblasts and pericytes (20 genes), some in both of these groups (15 genes) or other cell types (23 genes) of the healthy human hearts. The identity of genes per cell population are listed in Suppl. Table 1. Only expression profiles of the fibroblast-specific gene sets separated donors from HF patients as well as the whole 82 gene panel in a tSNE plot (Suppl. Fig. 1). In none of the plots, HF patients separated into sub-clusters. To increase the sensitivity for the detection of fibrosis related sub-cluster among the heart disease patients, we restricted expression profiling to the fibroblast specific genes with the highest variability within the data set using the projection score function of the Qlucore analysis program [10]. This highlighted 12 genes: seven collagen genes, fibrillin, the metalloproteinase inhibitor *TIMP3* and *TGFB1*, which are all classical fibrosis markers, as well as the monocyte attracting chemokine *CCL2*, and the pericyte-expressed membrane-bound angiotensin converting enzyme 2 (*ACE2*), were part of the panel.

**Supplementary figure 2:**
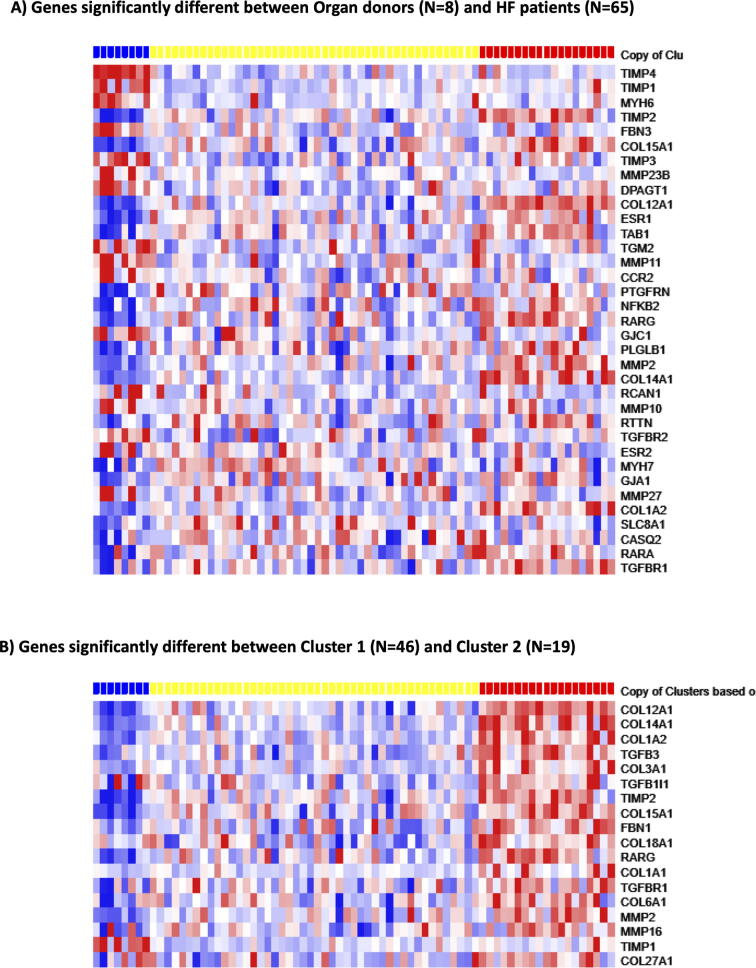


Applying these 12 CFB genes to tSNE analysis we distinguished donors from heart-failure patients, but the most prevalent distinction was a separation of the heart failure patients into two sub-clusters (Fig. 1D). Expression levels of all genes, except for *ACE2,* were higher in cluster 2 than in cluster 1. Of all genes tested, 35 were significantly different between HF patients and healthy controls and 18 between cluster 1 and cluster 2 (Suppl. Table 3; Suppl. Fig. 2). Testing only the 12 gene panel, all genes except CCL2, TIMP3 and ACE2 were significantly different between cluster 1 and cluster 2.

Since the 12-gene panel contains the major fibrillar collagens, fibrosis is suggested to be more severe in cluster 2 patients (Fig. 1E). Interestingly, the large majority of patients in cluster 2 (84 %) were either diagnosed with ischemic heart failure or had ECG readings indicating prior infarcts, suggesting ischemic disease. On the other hand, only less than half of the cluster 1 patients (46 %) showed ischemic HF or indication of prior infarction determined by ECG (Fig. 1D). Also, female, diabetic, and overweight patients appeared more prevalent in cluster 2 (Fig. 1F).

These data show that an expression analysis of 12 cardiac fibrosis-related genes is quite effective to distinguish heart failure patients from donors, and they suggest an association between ischemia and severe fibrosis, while diabetes, high BMI, and gender were less strongly associated with increased expression of these 12 fibrosis related genes.

### Macrophages are abundant in primary cardiac fibroblasts cultures

**Fig. 2:**
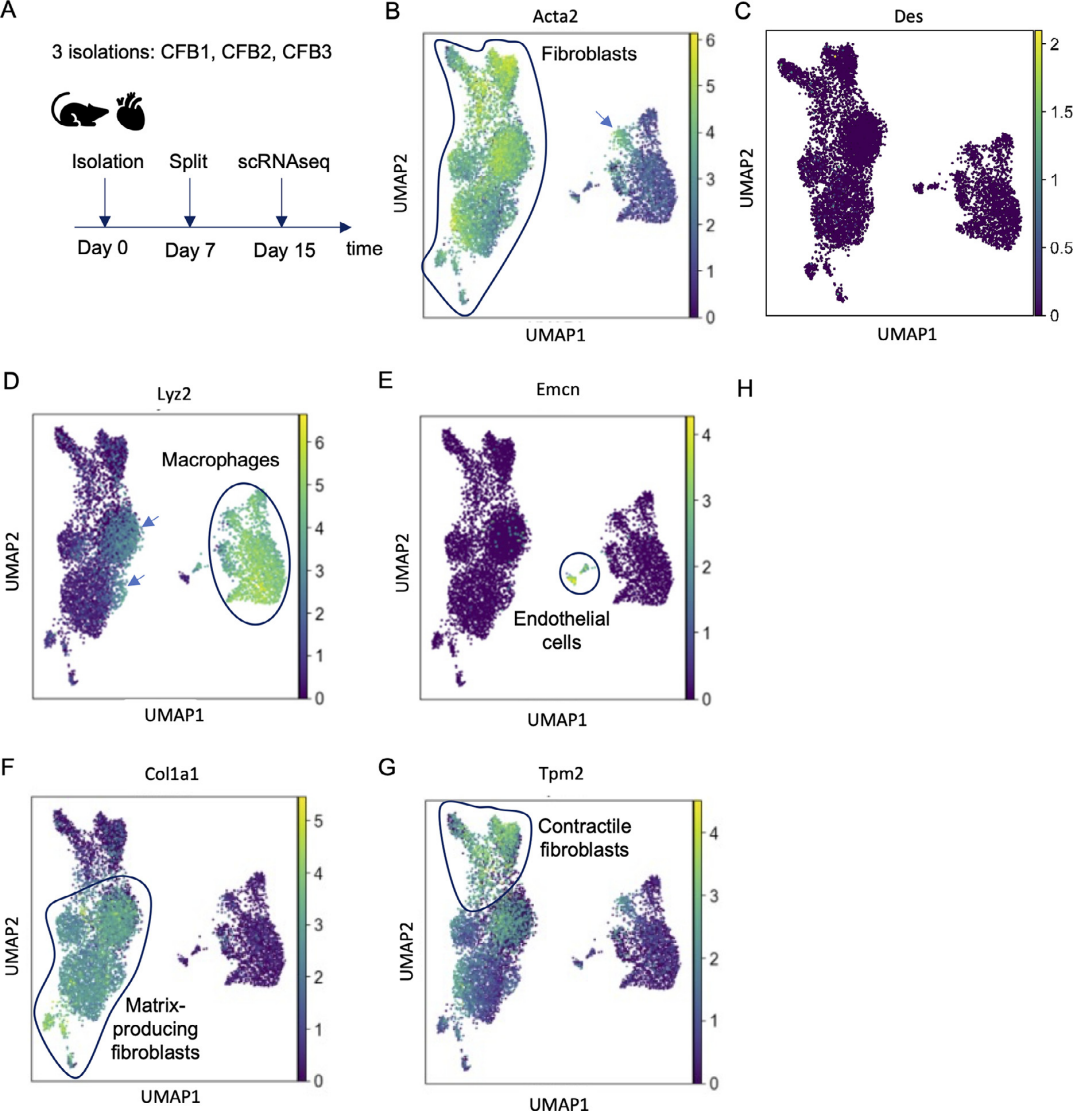
Different cell types in preparations of cardiac fibroblasts from mice cultured for 15 days *in vitro*. A) Experimental set up. B-G) UMAP plots for expression of indicated genes (color code indicates expression level).

**Supplementary figure 3:**
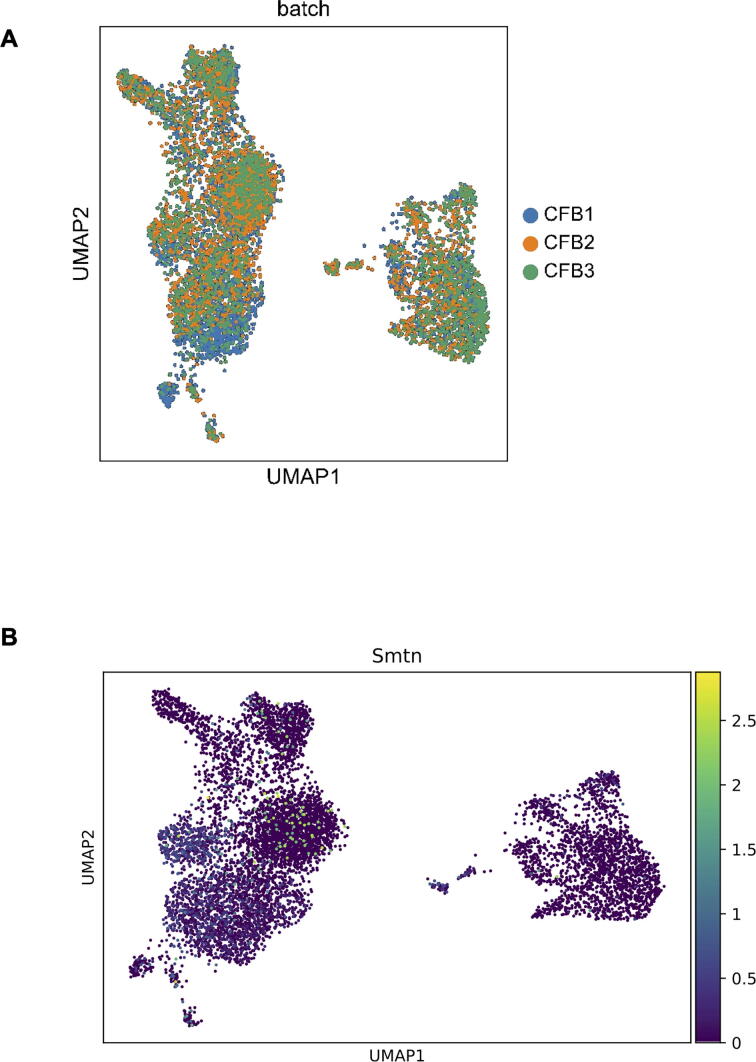


To investigate heterogeneity of primary cardiac fibroblasts (CFB) in more detail, we investigated *in vitro* activation of CFB from the murine left ventricle cultured on plastic for 15 days and performed scRNAseq (Fig. 2A). A UMAP plot was generated to visualize the scRNAseq data in two dimensions and showed high similarity of the three biological replicates after batch correction using Seurat (Suppl. Fig. 3A).

**Supplementary figure 4:**
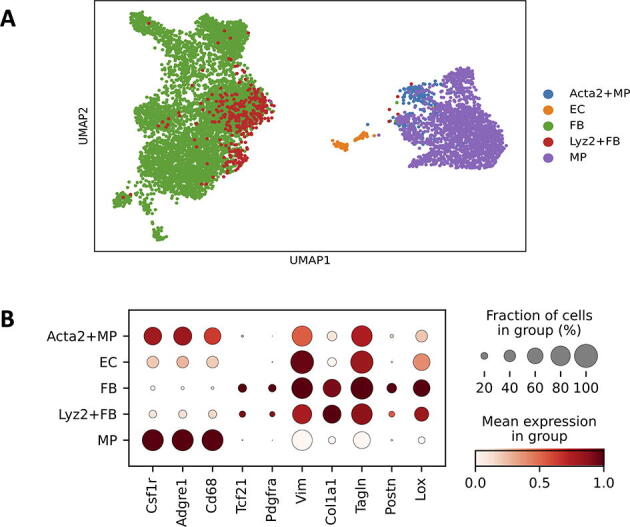


UMAP analysis clearly separated the cells into three distinct populations. The largest population expressed *Acta2* (Fig. 2B; 74.3 %), which is the classical marker of myofibroblasts and smooth muscle cells *in vivo*. Although all mesenchymal cells will start expressing *Acta2* when cultured *in vitro* on a regular tissue culture dish, the low expression of other smooth muscle cell markers such as *Des, Smtn, or Myh11* (Fig. 2C; Suppl. Fig. 3B, 4) indicated that these cells likely represent cardiac fibroblasts. Around 24.9 % of the cultured cells showed high expression *Lyz2* (Fig. 2D), a gene encoding lysosomal protein typically expressed in phagocytosing cells such as macrophages. Surprisingly, some cells in the macrophage population expressed the fibroblast marker *Acta2* (Fig. 2B, arrow) and some fibroblasts the macrophage marker *Lyz2* (Fig. 2D arrows). These double positive populations could also be observed when parameters were adjusted to more stringently eliminate potential doublets. *Lyz2 +* fibroblasts showed strong expression of the fibroblast markers *Vim, Col1a1, Tagln*, and *Lox*, but only weak expression of the macrophage markers *Csf1r, Adgre1*, and *Cd68* (Suppl. Fig. 4). Yet, the expression of these macrophage markers was higher than in *Lyz2*- fibroblasts. On the other hand, *Acta2* + macrophages showed expression of macrophage and fibroblast markers (Suppl. Fig. 4).

In addition, a small population of cells with high expression of endothelial markers such as *Emcn* was identified (Fig. 2E; 0.89 %).

These data reveal that the standard isolation procedure of primary CFB results in a mixed culture of fibroblasts and macrophages.

### Two main subtypes of activated cardiac fibroblasts govern extracellular matrix production and contraction

**Supplementary figure 5:**
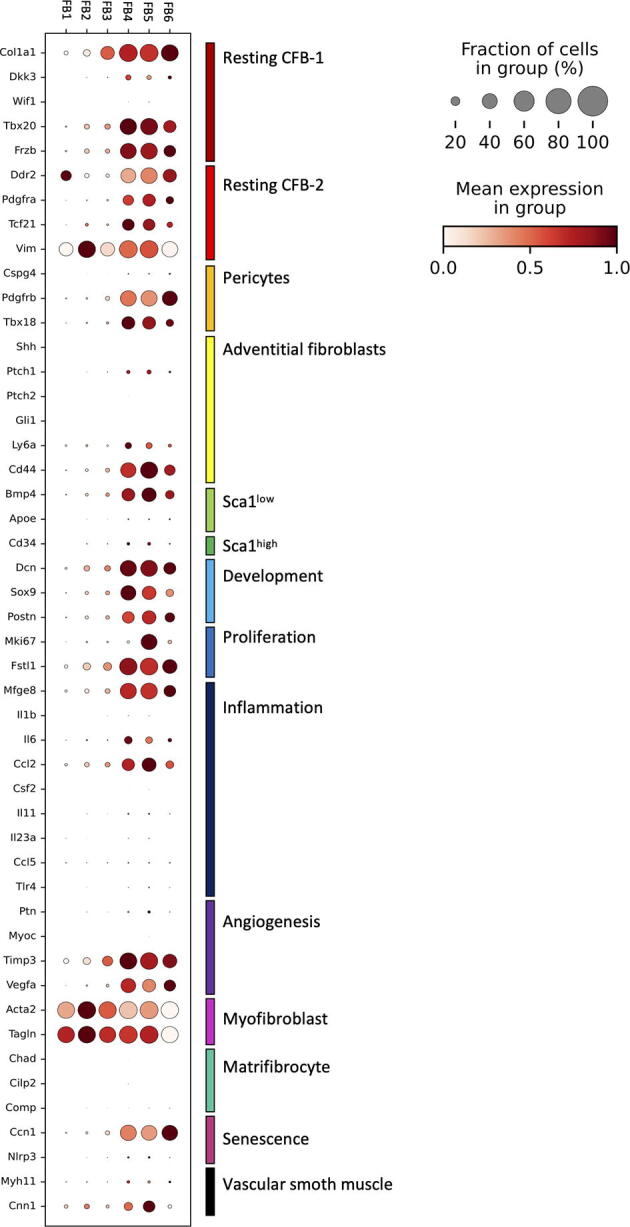


Cardiac fibroblasts are the main producers of extracellular matrix in the heart and are often identified by the expression of collagen type I (*Col1a1*, *Col1a2*) and III (*Col3a1*), the main types of collagen in the heart. Interestingly, 76 % of the CFB population showed a matrix-producing phenotype, characterized by high levels of *Col1a1* and lower levels of *Acta2* (Fig. 2F). The remaining fibroblasts (24 %), on the contrary, displayed high levels of *Acta2* and low amounts of *Col1a1*. The latter population was further characterized by expression of genes involved in Ca^2+^ handling and contraction such as *Tpm2* (Fig. 2G), indicating that *in vitro* culture of CFB results in two major subtypes of activated fibroblasts, a contractile one and a matrix producing one. The pericyte marker *Cspg4* was absent in all CFB (Suppl. Fig. 5).

These data challenge the general perception that cardiac fibroblasts uniformly become highly contractile and highly collagen producing myofibroblasts when culturing *in vitro*. Instead, these properties appear to be rather divided among two major subpopulations.

### Distribution of known cardiac fibroblast markers across sub-groups

**Fig. 3:**
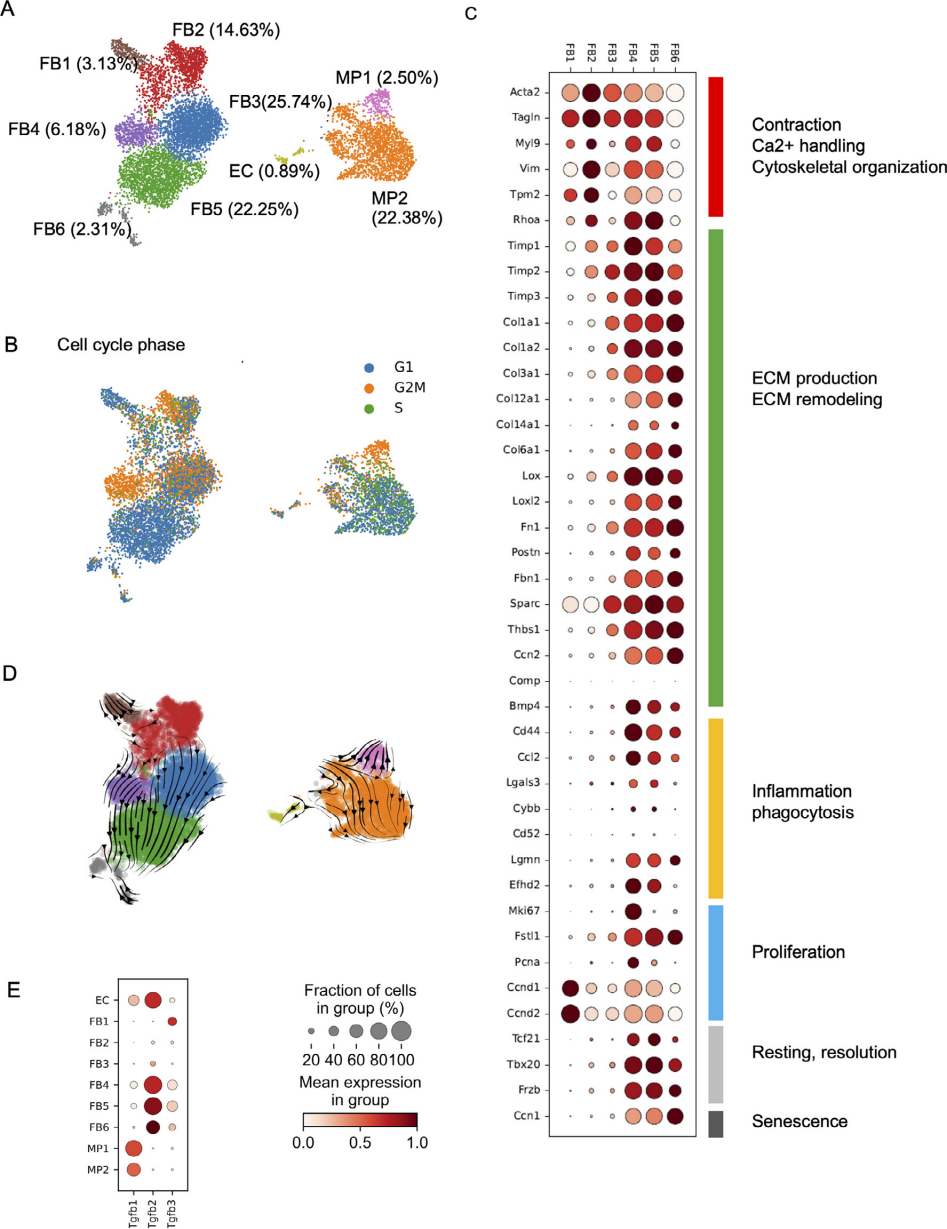
Different subpopulations of cardiac fibroblasts from mice cultured for 15 days *in vitro*. A) UMAP plot with colors indicating different indicated subpopulations in preparations of cardiac fibroblasts from mice culture for 15d *in vitro*. (Numbers in brackets indicate size of subpopulation). B) UMAP plot with colors indicating cell cycle phase of cells. C) Dot plot representing expression of indicated genes in fibroblast subpopulations FB1-FB6. ED) RNA velocity plot with arrows indicating the calculated direction of development. E) Dot plots representing expression of Tgfb1/2/3 in different subpopulations of the cultured primary cells (EC: endothelial cells; FB1-FB6: fibroblasts; MP1-MP2: macrophages).

Further analysis of the fibroblasts revealed 6 subpopulations (FB1-FB6; Fig. 3A) with FB3 (25.7 %) and FB5 (22.3 %) as the largest ones. Cells in sub-group FB4 were almost entirely in G2/M, suggesting most likely a transient stage, as mammalian cells normally exit the cell cycle at G1 (Fig. 3B).

**Supplementary figure 6:**
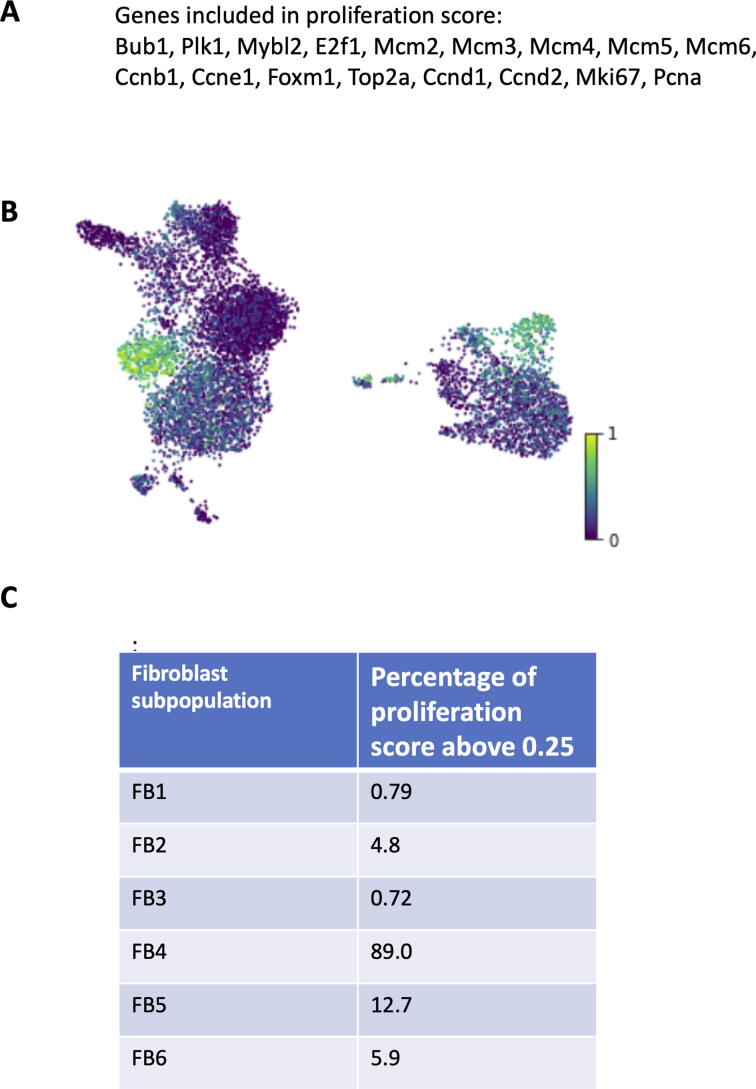


To gain a clearer understanding of phenotypic and functional properties of the fibroblast subpopulations, we analyzed them for the expression of well-known cardiac fibroblast markers and novel cardiac fibroblast markers recently identified by scRNAseq of mouse hearts [[8], [11]] (Fig. 3C; Suppl. Fig. 5). Contraction and cytoskeleton related genes such as *Acta2* and *Tpm2* were highest in FB2 and largely absent in FB6. Several contraction related genes (*Tagln*, *Myl9*, *Vim*, *Tpm2*, *Rhoa*) showed lower levels in FB3 than in FB4. Conversely, nearly all ECM related genes increased from FB1 to FB6. FB4 and FB5 were quite similar with respect to contraction and ECM related gene expression, but showed clear differences with respect to cell cycle phase. Inflammation and phagocytosis related genes (*Cd44*, *Ccl2*, *Efhd2*) displayed highest amounts in FB4. Proliferation markers showed a heterogenous expression pattern. While *Ccnd1* and *Ccnd2* strongly peaked in FB1, the widely used markers *Mki67* and *Pcna* were most strongly expressed in FB4. To obtain a clearer picture we made a score of 17 proliferation related genes including *Ccnd1*, *Ccnd2*, *Mki67* and *Pcna* with equal weighting (Suppl. Fig. 6A). This analysis indicated FB4 as the subpopulation with the highest percentage of proliferating cells, while FB1 and FB3 showed the lowest amounts of proliferating cells (Suppl. Fig. 6B, C). Cell proliferation occurred also in the strongly ECM producing cell populations FB5 and FB6. The senescence related marker *Ccn1* was hardly present in FB1-3, but increased strongly from FB4 to FB6, suggesting that FB6 might be a activation end point. Three markers associated with resting fibroblasts and resolution of fibrosis during wound healing (*Tcf21, Tbx20, Fzb*) were very low in FB1-FB3 and high in FB4-FB6.

Checking expression of cardiac fibroblast subpopulation markers described earlier [11] in FB1-FB6, we noted high expression of the resting cardiac fibroblasts marker Vim in FB2 (Suppl. Fig. 5). Other markers for resting cardiac fibroblasts, pericytes, and development were particularly high in FB4 and FB5. Inflammation or matrifibrocyte markers were mostly not detectable.

Assuming a gradual change in the expression of cardiac fibroblast related genes during activation, a development path from FB2 to FB1 and from FB2 to FB6 appears possible.

### RNA velocity analysis suggests branched activation process

To understand potential cell activation dynamics, we performed RNA velocity analysis. This analysis suggested a branched activation path from FB2 to FB3 and FB4 (Fig. 3E) and from FB3 and FB4 to FB5, respectively. Both, FB4 and FB5 can develop into FB6. A path from FB2 and FB1 is suggested by the strong arrows within the FB1 population (Fig. 3D). However, clear arrows connecting FB1 and FB2 are missing.

### Macrophages are the major source for TGFβ1 in cell culture

In addition to mechanical stress, TGFβ signaling is accepted as one of the main drivers of myofibroblast activation, and inhibition of this pathway significantly reduces *Acta2* and *Col1a1* gene expression in primary mouse cardiac fibroblasts *in vitro* [8]. Interestingly, in our primary culture system macrophages were the main producers of TGFβ1, while TGFβ2 and TGFβ3 were expressed by fibroblasts (Fig. 3E). Thus, the presence of macrophages in primary cardiac fibroblast cultures may be an important contributor to cardiac fibroblast activation in primary cell culture.

### The most specific markers for each sub-group include novel cardiac fibroblast markers

**Fig. 4:**
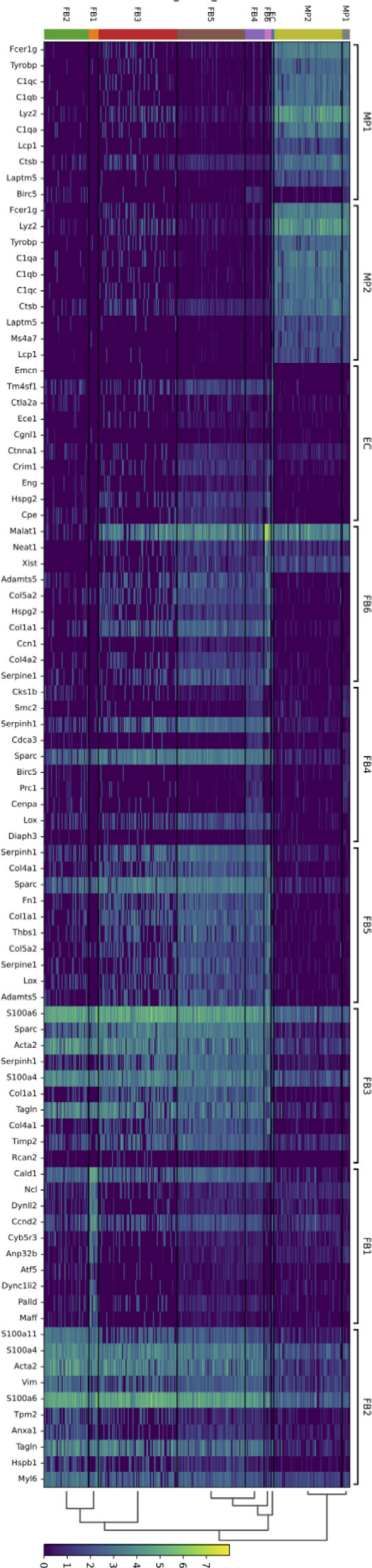
Heat map of the top 10 expressed genes in each subpopulation identified in the preparations of cardiac fibroblasts from mice cultured for 15d *in vitro*. (FB1-FB6: fibroblasts; MP1-MP2: macrophages; EC: endothelial cells; color code indicates expression level).

To further characterize the cardiac fibroblast sub-groups, we examined which genes were most highly expressed in each sub-group compared to the other groups (Fig. 4). Interestingly, some of these markers were shared between the subpopulations (FB2/FB3: Acta2, Tagln, S100a4; FB3/FB5: Col1a1, Serpinh1; FB4/FB5: Lox, Serpinh1, Sparc; FB5/FB6: Adamts4, Col1a1, Col5a2, Serpine1). Since shared markers might indicate a close developmental relationship, these data support that both FB3 and FB4 can develop into FB5, which then develop further to FB6. Caldesmon1 *(Cald1)* and *Malat1* were found to be relatively specifically expressed in FB1 and FB6, respectively, and could be useful single markers for these populations. Malat1 is a lncRNA that already earlier had been suggested to mediate cardiac fibrosis [12]. *Cald1* is an actin- and calmodulin-binding molecule crucial for the regulation of smooth-muscle and non-muscle cell contraction, which was reported to be upregulated in liver fibrosis [13]. Highest expression in the macrophage populations displayed genes of complement proteins.

### Subpopulation FB6 corresponds best to the fibrosis expression profile detected in heart failure

Comparison of the characteristic 12-gene expression pattern observed in heart failure patients with the expression pattern observed in activated fibroblast subsets *in vitro* with respect to expression level revealed a very high overlap with FB6, the population with the highest amount of ECM production (Suppl. Fig. 7A). Calculating the Pearson correlation between the normalized expression of the 12 genes in HF patients and in fibroblast subsets identified *in vitro* only FB6 revealed a positive correlation coefficient (Suppl. Fig. 7B). This similarity indicates that the FB6 population might be a good model for the activated fibroblasts present in heart failure patients, despite the differences between *in vitro* and *in vivo* conditions for example with respect to stiffness.

These data suggest that size, fate, and function of the FB6 subpopulation of primary cardiac fibroblast cultures could be used as a surrogate model for screening and investigating drugs regulating fibrosis associated with heart failure.

### Comparison of sub-type marker gene expression to bulk RNA gene expression at different time points after myocardial infarction

The previous analysis revealed that, at least *in vitro*, cardiac fibroblast activation is characterized by changes in subpopulations sizes rather than by a parallel change in gene expression of all fibroblasts. If this is relevant also for fibrosis *in vivo*, we would expect firstly, that the top marker genes of FB1 to FB6 would be highly relevant for clustering of fibroblasts during a fibrotic disease process, and secondly, that certain subpopulations might dominate at a given time point during a fibrotic disease process. To test these predictions, we took advantage of a bulk RNAseq data set of mouse hearts at different time points after myocardial infarction (MI), published earlier by the Molkentin group [14].

**Fig. 5:**
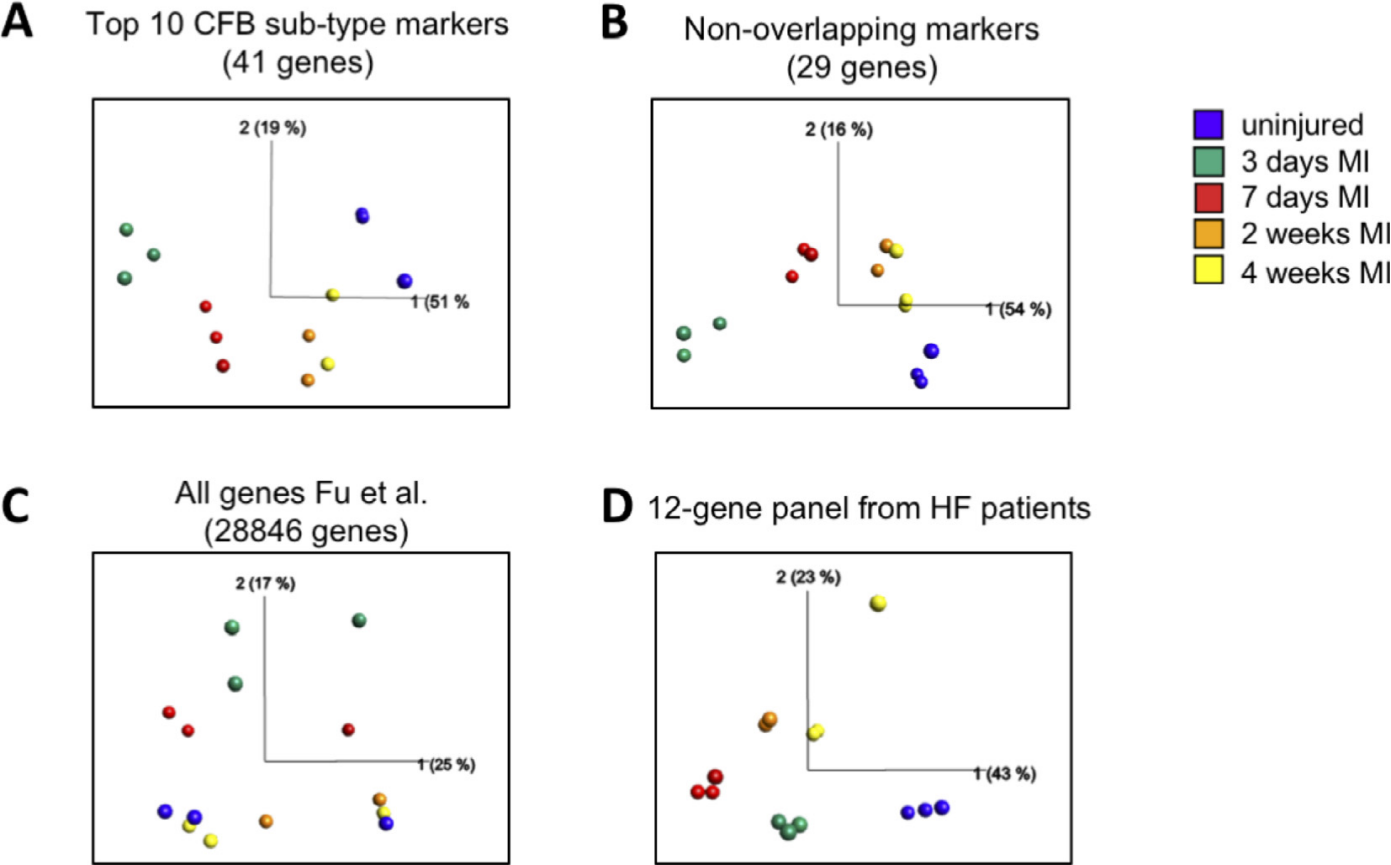
Fibroblast subpopulation marker cluster well different stages of a murine myocardial infarct model. A-D) PCA of indicated fibroblast subpopulation markers or all genes (C) results in clustering of different disease stages of murine myocardial infarct model published by Fu et al. (2018). Colors indicate disease stages. The percentage of variation represented by the PC is indicated.

Performing a principal component analysis (PCA) of the top 10 marker genes for FB1 to FB6, as far as present in the MI data set, or with only the non-overlapping markers resulted in distinct separation of the different disease stages (Fig. 5A, B). The clustering was better compared to a PCA conducted with all 28 846 genes reported in the study by Fu et al. (Fig. 5C), suggesting that our marker gene set focuses on genes with high relevance for fibrosis. An even more distinct separation of samples according to their disease stage was achieved with the 12-gene profile identified in the heart failure patients (Fig. 5D), supporting this notion. Multi group comparison test (ANOVA)test identified 2361 differentially expressed genes in the MI model. Performing a hyper-geometric test we found that an overlap of 24 genes of the 41 marker gene set and of 7 genes of the 12 gene-panel with the differentially expressed genes, which was both highly significant (p (41marker set) = 6.0 exp −4; p (12-gene panel) = 1,2 exp-5) and extremely unlikely to be explained by chance.

**Fig. 6:**
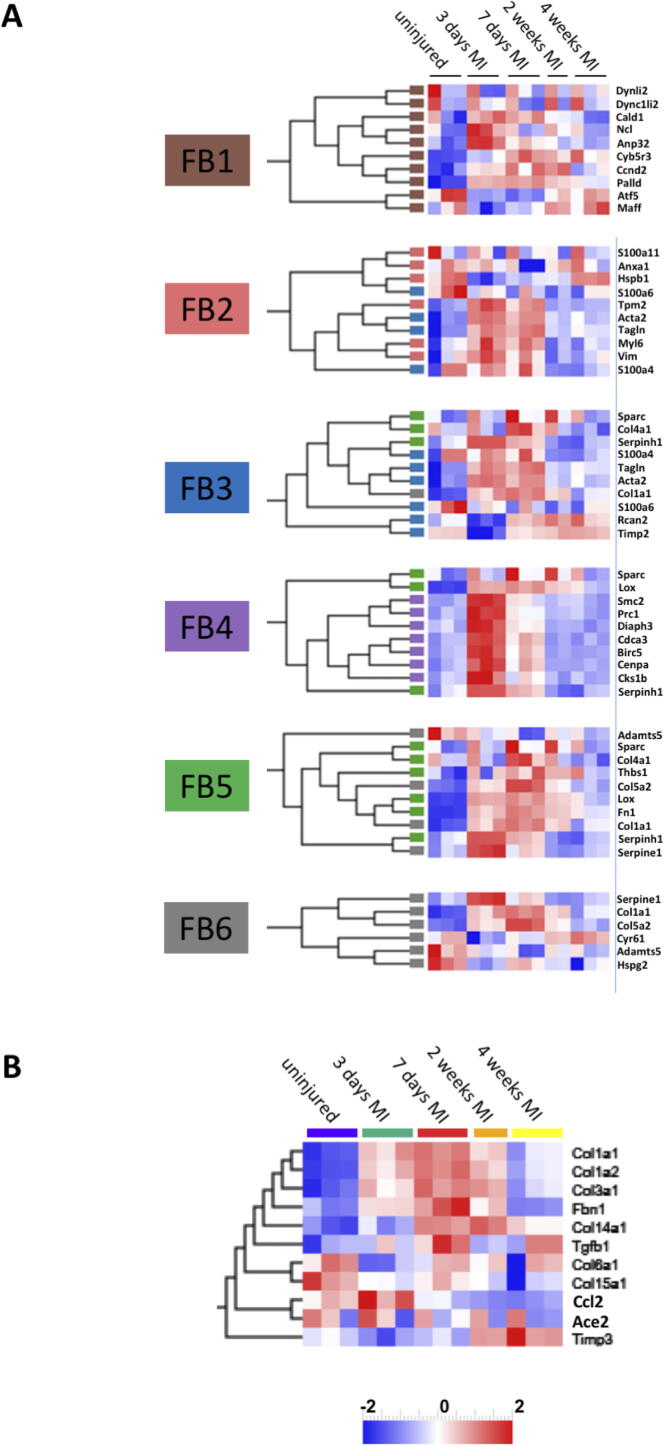
Stage specific expression of fibroblast marker genes in a murine myocardial infarct model. A) Heatmap for top10 marker genes for FB1-FB6. B) Heatmap for 12-gene HF expression profile. Color indicates expression level relative to mean expression of a gene across samples, red color indicates increased and blue color decreased expression. (For interpretation of the references to color in this figure legend, the reader is referred to the web version of this article.)

Analyzing the bulk expression data at the different time points for the subpopulation markers we found a high contribution of FB4 markers at 3d after MI, which strongly decreased at 7d and was back to levels of uninjured hearts after 2w (Fig. 6A). FB5 and FB6 markers peaked at 7d, suggesting a switch to more ECM producing subpopulations from 3d to 7d. Interestingly, FB2 and FB3 markers were equally present 3d and 7d after MI, indicating that size of the less activated populations is not simply inversely coupled to that of the more activated ones. As expected, due to the similarity of the 12-gene profile with FB6 markers, the 12-gene profile scored highest 7d after MI (Fig. 6B).

These data suggest that the fibroblast subpopulations observed *in vitro* might be of relevance *in vivo*.

## Discussion

In this study we identified a panel of 12 fibrosis-related genes whose expression distinguishes hearts from HF patients from donor hearts. It consists of genes for the fibrillar collagens I and III (COL1A1,COL1A2,COL3A1), the microfibrillar collagen VI (COL6A1), the TGFβ induced FACIT collagens XII and XIV (COL12A1,COL14A1), and the non-fibrillar, basement membrane associated collagen XV (COL15A1). Collagen 15 has earlier been suggested to maintain the basement membrane integrity of cardiac endothelial cells. In addition, TGFB1, and the TGFβ induced genes fibrillin (FBN1), which controls TGFβ storage in the ECM, and TIMP3, an inhibitor of matrix-degrading proteases, are part of the panel genes. Finally, the macrophage recruiting chemokine CCL2, and ACE2, a receptor of the angiotensin converting enzyme family, belong to the 12-gene profile. Interestingly, hydroxyproline content, which is a measure for fibrillar collagen, was less useful for distinguishing HF hearts from donor hearts than the expression of the 12-gene panel, suggesting that the non-fibrillar collagens, TGFB1, CCL2, and TGFβ1-induced genes included in the panel are markers for HF fibrosis independent of the fibrillar collagens. This indicates that overexpression of classical fibroblast-specific fibrosis markers is the common denominator of all HF hearts compared to the donor hearts irrespective of differences in clinical parameters of the HF patients, such as diabetes and obesity. However, subclustering of the HF hearts was not very strong, pointing to diversity in fibrosis in HF. Ischemic heart disease was enriched in a subpopulation distinguishable by the 12-marker gene panel in a tSNE plot. Besides, however, no strong sub-clustering of HF hearts was observed in relation to the clinical parameters recorded (gender, high BMI, and diabetes). This indicates that differences in fibrosis among patients sharing a specific clinical parameter are similar to differences in fibrosis between these patient groups.

In this study, only 82 fibrosis related genes were profiled. A full transcriptome screen might reveal more subtle differences in the matrix of failing hearts vs donor hearts and with respect to clinical parameters. Also, enrichment for fibroblasts by flow cytometry might facilitate the recognition of fibrosis subtypes.

The importance of ACE2 in the panel is not clear, since most reports indicate an antifibrotic effect. Future experiments need to validate this finding and identify also the cell type expressing ACE2 in HF hearts. ACE2 expression was recently reported to be increased in diabetes and obesity [15]. In addition, SARS-CoV-2 is entering cells via ACE2 and even mild SARS-CoV-2 infections were found to increase the risk of HF [16]. Thus, the identification of ACE2 gene expression as helpful to distinguish HF patients from healthy individuals in our study fits well in this context and supports further investigations on ACE2 function in HF.

*In vitro* activation of primary cardiac fibroblasts from mice and their analysis by scRNAseq resulted in clear subpopulations. Expression of the 9 of the 12 marker genes characterizing HF hearts was highest in the non-dividing, most activated FB6 population, suggesting that the *in vitro* activation is modelling the activation of fibroblasts in HF patients at least to some extent, despite the unphysiologically high stiffness of the tissue culture plastic and the artificial culture conditions. Probably, the “contamination” of the primary cardiac fibroblast preparation with TGFβ1 producing macrophages contributed to the efficient activation of cardiac fibroblasts during culture.

Fibroblasts with macrophage markers and macrophages with fibroblast markers have previously been described in murine skin wounds [17]. We show here, that these non-classical fibroblasts and macrophages can also be observed during *in vitro activation* of murine cardiac fibroblast, indicating that their presence is not restricted to skin wounds *in vivo.* The similarity in this unusual populations suggests that fibroblasts independent of their tissue origin and their context show rather similar activation.

The current study, which to our best knowledge is the first report on single cell transcriptomics of primary cardiac fibroblasts, gives several novel insights into this widely used model system. Initially, we expected to see *in vitro* a linear development of non-activated, primary mouse cardiac fibroblasts to contractile, ECM producing myofibroblasts. Instead, our analysis indicated the presence of 6 subpopulations and a clear distinction between a proliferating, contractile myofibroblast (FB2) and an equally proliferating, ECM producing matrix fibroblast (FB6), which later might become senescent.

Moreover, RNA velocity analysis suggested that FB2 can activate into at least two different subpopulations: the poorly proliferating FB3 and the highly proliferating FB4. Both, FB3 and FB4, develop into FB5, which then develops into FB6. Fibroblast activation, therefore, appears at least *in vitro* not to be a gradual process which affects all cells simultaneously. It appears to be better described as transitions between distinct subpopulations. For that reason, altered bulk expression might reflect mainly changes in subpopulation sizes and not of the expression levels in all fibroblasts in culture. This has consequences for the analysis of fibroblast activation experiments as well as for drug development. Preferentially, for analysis of fibroblast development *in vitro*, markers should be chosen which are characteristic for the respective fibroblast subpopulations.

It remains to be tested whether the same fibroblast subpopulations FB1-FB6 will be identified *in vivo*. Earlier, a matrifibrocyte population was described to occur in mice 10 d after a myocardial infarct. These cells express chondrocyte and osteoblast markers that might be optimal to support the mature scar tissue [14]. We could not identify this population in the *in vitro* activated cardiac fibroblasts after 15d in culture. Furthermore, we failed to detect the reparative Cthrc1 + cardiac fibroblast subpopulation, which was reported to arise after myocardial infarct in mice [18]. These alterations might be due to the differences between the *in vivo* and the *in vitro* environment. Yet, also suboptimal clustering of populations has to be considered. Comparision of our fibroblast subpopulations with bulk RNAseq data of myocardial infarct in mice, indicated at least some similarity of the expression pattern of specific fibroblast subsets *in vitro* with certain stages of MI *in vivo*. However, also differences were observed. For example, Col15a1 appeared to be downregulated during MI, but upregulated in HF, particularly in cluster 2 patients. Timp3 was upregulated at late stages of MI, but rather downregulated in HF patients. Species specific differences or differences between the pathological processes occurring in human HF patients and in the murine MI model might explain these findings.

Finally, fibrosis therapy aims to reduce excessive ECM production. The data provided by this study suggest that drug development should focus on preventing activation of FB2 to matrix producing fibroblasts and on promotion of senescence or apoptosis of FB5 or FB6 matrix producing fibroblasts.

## Experimental procedures

### Patient samples and analysis

Myocardial samples from 10 organ donors where the heart could not be used for transplantation were used as controls (mean age 43.3 ± 14.7 years, 6 females). Hearts removed during heart transplant of 65 patients with end-stage heart failure (mean age 52.5 ± 15.0 years, 23 females, 26 ischemic heart failure) were acquired at the University of Kentucky (published protocol [19]). (detailed description of tissue harvesting protocol in [19]). Clinical characteristics of the patients and organ donor controls are listed in Suppl. Table 2. During tissue collection, the left ventricular myocardium is separated into three transmural regions and frozen in liquid nitrogen. Mid-wall left ventricular samples were divided for examination by histology or protein and mRNA quantification. All procedures were approved by The University of Kentucky Institutional Review Board and informed consent (IRB #46103) was given by subjects, or their legally authorized representatives. Collagen content was determined by hydroxyproline measurement and visualized by picrosirius staining of tissue sections. RNA was isolated using Trizol (Invitrogen) and cDNA synthesized using a High Capacity cDNA Archive Kit (Applied Biosystems). mRNA was measured using a custom made nCounter reporter probe for 82 fibrosis associated genes and the Nanostring nCounter System. Transcription levels of fibrosis-related genes were measured using a custom-made Nanostring panel of fibrosis related genes. Transcript levels were normalized against the following five reference genes: *GAPDH*, *GUSB*, *PGK1*, *POLR2A*, and *RPLP0*. Data were analyzed using Qlucore Omics Explorer (Qlucore AB, Lund, Sweden) and corrected for age and grouping caused by reference genes. The projection score function of Qlucore was used for selecting optimal gene subset via variance filtering, which aims to calculate the non-random variance [20].

### Cardiac fibroblasts

Three rounds of cardiac fibroblast isolation were performed, each time from two adult female mice (C57BL/6, Taconic, Denmark). Left ventricles were dissected and cut into small pieces whereafter the tissue was subjected to digestion as previously described [21]. Cardiac fibroblasts were allowed to attach to a T75 tissue culture flask for 30 min at 37 °C and 5 % CO2 (passage 0) before aspirating the solution and replacing the media with DMEM containing 10 % fetal bovine serum (FBS). Cells were passaged (passage 1) after 7 days when they reached ∼ 80 % confluency and plated into 6-well tissue culture plastic plates. After a total of 15 days, the cardiac fibroblasts were collected for single cell analysis. Mice were kept in an AAALAC (Association for Assessment and Accreditation of Laboratory Animal Care)-accredited animal house under specific pathogen-free conditions. Licenses for breeding were obtained from the Danish Administration for Animal Experiments.

### Single-cell RNA sequencing

RNA isolation (GenElute Mammalian Total RNA Miniprep Kit, Sigma), cDNA synthesis (TaqMan Reverse Transcription Reagents kit, #N8080234, Applied Biosystems) were carried out according to the instructions of the manufacturer. scRNAseq was performed using the Chromium Single Cell 3ʹ Reagent Kit v3 and the Chromium i7 Multiplex kit (10x Genomics, Pleasanton, USA) following the manufacturers’ instructions. Sequencing was performed by BGO Tech Solutions (Hongkong, China) Co using the HiSeq Sequencing platform.

To estimate developmental directions of cells, we measured RNA velocity using the Python package scVelo [22]. Firstly, we extracted spliced and unspliced reads using the velocyto pipeline (https://velocyto.org), and converted the results to an AnnData object for downstream analysis. Secondly, we merged the three samples by removing batch effects. Then, 52 doublets were removed from 8177 cells using Scrublet [23]. Finally, we followed the suggested pipeline of scVelo, including preprocessing, estimating velocities, constructing velocity graph and projecting velocities on low-dimensional space.

We filtered genes with spliced and unspliced counts of less than 20 and normalized expression by original counts and log transformed the data. Then, we estimated RNA velocities using the default mode “stochastic”. Next, the velocity graph was constructed by computing correlations between velocities and potential cell state transitions. To visualize RNA velocity, we embedded initial data in a 2D UMAP [21] space and clustered cells by Louvian algorithm [23]. To identify cell populations, we used t-tests to obtain top 10 marker genes of each cell population. Finally, the RNA velocities were projected into 2D UMAP embedding.

### Statistics

Statistics were performed using software from Qlucore Omics Explorer (Qlucore AB, Lund, Sweden) and GraphPad Prism 8.2.0. We calculated q values using Benjamini-Hochberg.

## Funding

KMH has received funding from the European Union’s Horizon 2020 research and innovation programme under the Marie Sklodowska-Curie grant agreement No 795390. KJW is supported by The 10.13039/501100000008Novo Nordisk Foundation Center for Stem Cell Biology [NNF17CC0027852] and the Independent Research Fund Denmark [0135-00243B]. The work was also supported by Independent Research Fund Denmark (DFF-0199-00001B) to AL.

The authors acknowledge support from the Gill Cardiovascular Biorepository at the University of Kentucky and from the patients, organ donors, and families that donated samples. Funding: NIH TR033173, HL133359, HL146676 (KSC), AHA TP135689 (KSC), and Penny Warren Award for Clinical and Translational Research.

## Author contributions

Kate Møller Herum (Concept, investigation, analysis, writing), Guangzheng Weng (Analysis, writing), Konstantin Kahnert (Analysis, writing), Rebekah Waikel (Resources), Greg Milburn (Resources), Autumn Conger (Resources), Paul Anaya (Resources), Kenneth S. Campbell (Resources), Alicia Lundby (analysis), Kyoung Jae Won (analysis), Cord Brakebusch (Concept, analysis, writing).

## Declaration of Competing Interest

The authors declare the following financial interests/personal relationships which may be considered as potential competing interests: Kate Herum reports financial support was provided by Marie Sklodowska-Curie grant (AU Horizon 2020). Kyoung Jae Won reports financial support was provided by Novo Nordisk Foundation. Alicia Lundby reports financial support was provided by Independent Research Fund Denmark. Kenneth S. Campbell reports was provided by National Institutes of Health. Kyoung Jae Won reports financial support was provided by Independent Research Fund Denmark.

## Figures and Tables

**Supplementary figure 7:**