# Supplementary material for: Cardiac fibroblast sub-types in vitro reflect pathological cardiac remodeling in vivo
Source: Matrix Biol Plus. 2022 Jun 6;15:100113. doi: 10.1016/j.mbplus.2022.100113 (PMC9198323; doi:10.1016/j.mbplus.2022.100113)
Supplement: Supplementary data 1 [file mmc1.docx]

**Suppl. Table 1:** **Clinical characteristics**

Body mass index (BMI), left ventricular ejection fraction (LVEF), left ventricular inner diameter at end-diastole (LVIDd), interventricular septum thickness at end-diastole (IVSd), left ventricular posterior wall thickness at end-diastole (LVPWd). Student’s *t*-test (two-tailed) was used to determined differences between organ donors and heart failure patients (*) and females and males (^†^). N is shown in brackets and denotes the number of patients where information was available.

|  | **Female** | |  | **Male** | |
| --- | --- | --- | --- | --- | --- |
|  | **Organ donor** | **Heart failure** |  | **Organ donor** | **Heart failure** |
| **Demographic** |  |  |  |  |  |
| Number of patients | 5 | 22 |  | 5 | 43 |
| Age, years | 41.3 ± 15.3 (5) | 51.2 ± 14.9 (22) |  | 42.0 ± 14.1 (5) | 53.6 ± 14.3 (43) |
| BMI, kg/m^2^ | 30.5 ± 4.3 (5) | 29.8 ± 6.2 (22) |  | 25.4 ± 2.5 (4) | 29.5 ± 4.1 (42) |
|  |  |  |  |  |  |
| **Echocardiography** |  |  |  |  |  |
| LVEF, % | 54.8 ± 18.9 (4) | 22.9 ± 9.0 (21) *** |  | 49.9 ± 13.6 (5) | 20.8 ± 4.5 (41)*** |
| LVIDd (mm) | 4.25 ± 0.88 (4) | 6.45 ± 1.04 (20) *** |  | 4.37 ± 0.11 (4) | 7.21 ± 0.99 (37) ***^,†^ |
| IVSd (mm) | 1.30 ± 0.60 (2) | 0.88 ± 0.13 (19) |  | 0.90 ± 0.08 (3) | 0.92 ± 0.18 (36) |
| LVPWd (mm) | 1.3 ± 0.5 (2) | 0.93 ± 0.16 (19) |  | 0.93 ± 0.04 (3) | 0.92 ± 0.17 (36) |
|  |  |  |  |  |  |
| **Comorbidities** |  |  |  |  |  |
| Diabetes | 1 (5) | 9 (21) |  | 0 (3) | 14 (21) |
|  |  |  |  |  |  |
| **Medications** |  |  |  |  |  |
| ACE inhibitor | 0 (4) | 10 (22) |  | 0 (3) | 19 (39) |
| ARB | 0 (4) | 7 (22) |  | 0 (3) | 9 (39) |
|  |  |  |  |  |  |
| **Heart failure type** |  |  |  |  |  |
| Ischemic |  | 5 (21) |  |  | 21 (42) |
| Non-ischemic |  | 16 (21) |  |  | 21 (42) |
