# Supplementary material for: Cardiac fibroblast sub-types in vitro reflect pathological cardiac remodeling in vivo
Source: Matrix Biol Plus. 2022 Jun 6;15:100113. doi: 10.1016/j.mbplus.2022.100113 (PMC9198323; doi:10.1016/j.mbplus.2022.100113)
Supplement: Supplementary data 2 [file mmc2.docx]

**Suppl. Table 2:** 82 genes measure in human myocardium

| **CM** | **CFB** | **Both** | **Other** |
| --- | --- | --- | --- |
| ATP2A2 | ACE2 | BACE1 | ACE |
| CASQ2 | CCL2 | CTNNA1 | AGER |
| COL21A1 | CCR2 | CTNNB1 | AGTR2 |
| COL4A5 | COL12A1 | HACE1 | DPAGT1 |
| CTNNBIP1 | COL14A1 | IL6R | FBN3 |
| CTNNBL1 | COL15A1 | MEF2D | GPER1 |
| DES | COL18A1 | MMP2 | MCOLN1 |
| ESR2 | COL1A1 | MMP23B | MMP10 |
| FBN2 | COL1A2 | PTGFRN | MMP11 |
| GJA1 | COL27A1 | RhoA | MMP27 |
| HSPA1A | COL3A1 | TAB1 | NFKB2 |
| LACE1 | COL6A1 | TGFBR1 | PLGLB1 |
| MYH6 | ESR1 | TGFBR2 | RARA |
| MYH7 | FBN1 | TGFBR3 | RARG |
| NKX2-5 | FGF2 | TIMP1 | SRF |
| NPPA | GJC1 |  | TGFA |
| NPPB | GUCY1A2 |  | TGFB1I1 |
| PPARGC1A | MMP16 |  | TGFB3 |
| PRKCZ | TGFB1 |  | TGFBR3L |
| RARB | TIMP3 |  | TIMP2 |
| RCAN1 |  |  | TIMP4 |
| RTTN |  |  | TNN |
| SLC8A1 |  |  | WAS |
| TGM2 |  |  |  |
