# Supplementary material for: Cardiac fibroblast sub-types in vitro reflect pathological cardiac remodeling in vivo
Source: Matrix Biol Plus. 2022 Jun 6;15:100113. doi: 10.1016/j.mbplus.2022.100113 (PMC9198323; doi:10.1016/j.mbplus.2022.100113)
Supplement: Supplementary data 3 [file mmc3.docx]

**Suppl. Table 3: Differentially expressed genes in HF patients**

1. **Genes significantly different between Organ donors (N=8) and HF patients (N=65)**

**Gene p-value q-value**

TIMP4 4.2237637736534116e-17 3.4634862943957973e-15

TIMP1 4.679677831172015e-09 1.918667910780526e-07

MYH6 1.0459473931877283e-08 2.858922874713124e-07

TIMP2 8.368492503291078e-08 1.715540963174671e-06

FBN3 3.818140081305443e-07 6.261749733340926e-06

COL15A1 1.8879978395483861e-06 2.211654612042395e-05

TIMP3 1.8412990057440758e-06 2.211654612042395e-05

MMP23B 2.6460153603549716e-06 2.712165744363846e-05

DPAGT1 3.4794928514819417e-06 3.17020459801688e-05

COL12A1 1.3742503214472502e-05 0.00010244411487152228

ESR1 1.2813736011519581e-05 0.00010244411487152228

TAB1 1.5805265118704278e-05 0.00010800264497781257

TGM2 1.720333670593974e-05 0.00010851335460669681

MMP11 4.960587865824282e-05 0.0002905487178554222

CCR2 0.00011542071633994478 0.000591531171242217

PTGFRN 0.00010889938014971968 0.000591531171242217

NFKB2 0.000178085802187428 0.0008112797655205053

RARG 0.0001765463031677477 0.0008112797655205053

GJC1 0.00022434350175899956 0.0009682193233809455

PLGLB1 0.00026962814380300206 0.0011054753895923084

MMP2 0.00036848682452023443 0.0014388533147932964

COL14A1 0.0005033358630400882 0.0018760700349676016

RCAN1 0.0005743634942099052 0.002047730718487488

MMP10 0.0006942446158151505 0.0023720024373684308

RTTN 0.0013817819085649827 0.004532244660093143

TGFBR2 0.001970798304162337 0.006215594651588908

ESR2 0.0026073267606964764 0.007770399061183453

MYH7 0.002653306996501667 0.007770399061183453

GJA1 0.0037096147991454794 0.01048925563896308

MMP27 0.004382830794163761 0.011979737504047612

COL1A2 0.005842870114484353 0.015455333851216677

SLC8A1 0.007827051879022256 0.02005682043999453

CASQ2 0.009520387626542594 0.02365672076898463

RARA 0.010601940912200339 0.025569386905894935

TGFBR1 0.011711935314885851 0.027439391309161138

1. **Genes significantly different between Cluster 1 (N=46) and Cluster 2 (N=19)**

**Gene p-value q-value**

COL12A1 1.3156371548908006e-10 1.0788224670104565e-08

COL14A1 5.631858485430026e-10 2.3090619790263107e-08

COL1A2 7.19695692322835e-08 1.9671682256824157e-06

TGFB3 2.022992788589695e-06 4.147135216608875e-05

COL3A1 6.748597589844359e-06 0.00011067700047344749

TGFB1I1 5.4584759359230014e-05 0.0007459917112428103

TIMP2 7.450282854317768e-05 0.0008727474200772242

FBN1 0.00012691090253954797 0.0013007116108643853

COL15A1 0.0001427610304607252 0.0013007116108643853

COL18A1 0.00028299084503910965 0.002146412841104281

RARG 0.00028793342990423285 0.002146412841104281

COL1A1 0.0005530731576384168 0.003779333243862515

TGFBR1 0.001064140542909765 0.00671227111681544

COL6A1 0.0016366851576246765 0.009150843576688626

MMP2 0.0016739348006137732 0.009150843576688626

MMP16 0.002651049336808982 0.013586627851146033

TIMP1 0.00578559788899477 0.02790700158221007

COL27A1 0.008602023823186698 0.0391869974167394
